# A molecular beacon-based approach for live-cell imaging of RNA transcripts with minimal target engineering at the single-molecule level

**DOI:** 10.1038/s41598-017-01740-1

**Published:** 2017-05-08

**Authors:** Mingming Chen, Zhao Ma, Xiaotian Wu, Shiqi Mao, Yantao Yang, Jie Tan, Christopher J. Krueger, Antony K. Chen

**Affiliations:** 1 Department of Biomedical Engineering, College of Engineering, Peking University, Beijing, 100871 China; 2 Peking-Tsinghua Center for Life Sciences, Peking University, Beijing, 100871 China; 3 Academy for Advanced Interdisciplinary Studies, Peking University, Beijing, 100871 China; 4 Wallace H Coulter Department of Biomedical Engineering, Georgia Institute of Technology, Atlanta, GA 30332 USA

## Abstract

Analysis of RNA dynamics and localization at the single-molecule level in living cells has been predominantly achieved by engineering target RNAs with large insertions of tandem repeat sequences that are bound by protein-based or oligonucleotide-based fluorescent probes. Thus, individual RNAs are tagged by multiple fluorescent probes, making them detectable by fluorescence microscopy. Since large insertions may affect RNA processes including trafficking and localization, here we present a strategy to visualize single RNA transcripts in living cells using molecular beacons (MBs) - fluorogenic oligonucleotide probes - with minimal target engineering. The MBs are composed of 2′-O-methyl RNAs with a fully phosphorothioate-modified loop domain (2Me/PS_LOOP_ MBs), an architecture that elicits marginal levels of nonspecific signals in cells. We showed that MBs can detect single transcripts containing as few as 8 target repeat sequences with ~90% accuracy. In both the nucleus and the cytoplasm, mRNAs harboring 8 repeats moved faster than those with 32 repeats, suggesting that intracellular activities are less impeded by smaller engineered insertions. We then report the first MB-based imaging of intracellular dynamics and localization of single long noncoding RNAs (lncRNAs). We envision the proposed minimally-engineered, MB-based technology for live-cell single-molecule RNA imaging could facilitate new discoveries in RNA research.

## Introduction

As the list of characterized RNA molecules and functions expands, visualizing the distribution and dynamics of various RNAs at the single-molecule level in living cells can add invaluable information regarding their physiological roles. Single-molecule fluorescence *in situ* hybridization (smFISH) is the gold standard for robust and versatile visualization of intracellular distributions of specific RNA molecules in fixed cells and tissues^1^. As individual fluorophores are difficult to detect when imaged under a widefield fluorescence microscope, one approach for achieving single-molecule sensitivity is to design fluorophore-tagged oligonucleotide probes complementary to unique sequences in the target RNA. When imaged, the multiple fluorophore-tagged probes hybridized to a single transcript are easily detected by conventional fluorescence microscopy as a bright spot representing a single RNA transcript. However, information pertaining to RNA dynamics cannot be easily acquired, as fixation is required during smFISH sample preparation.

Live-cell single-molecule RNA dynamics has been studied predominantly using engineered RNA molecules with multiple tandem repeats that are bound by specific protein or oligonucleotide probes. The most common approach is the MS2 system, in which a fluorescent protein (FP) fused to the coat protein of bacterial phage MS2 is co-expressed with an engineered RNA construct containing multiple tandem repeats of the MS2 binding sequence^2–4^. In this way, specific RNAs are labeled by multiple FPs through the MS2 protein-RNA interaction. Another approach employs molecular beacons (MBs)^5^, which are single-stranded oligonucleotide probes capable of forming a stem-loop structure with a fluorophore and a quencher at the two termini. In the absence of complementary target RNA, the complementary sequences flanking the loop domain anneal to form a stable stem, bringing the fluorophore and quencher together. Hybridization of the loop domain to target RNA disrupts the stem configuration, causing separation of the fluorophore from the quencher and restoration of its fluorescence. Currently, MBs are predominantly used in applications where detection of specific RNAs is based on ensemble fluorescence measurements. It has been demonstrated that MBs can also be used to image RNAs with single-molecule sensitivity when target RNAs are engineered with multiple repeats of a known MB target sequence^6^.

Although combining MBs with multiple repeats of an MB target sequence could be a widely utilized approach to image RNA dynamics, and compared with the MS2-FP system may offer the added benefits of smaller probe size, incorporation of a wider variety of fluorophores, and improved signal-to-background due to quenching when not bound to target, the tendency of MBs synthesized with conventional DNA or 2′-O-methyl RNA (2Me) backbones to generate false-positive signals in cells limits their utility in RNA research^7, 8^. False-positive signals are primarily detected in the nucleus, arising as a result of nuclease degradation and/or nonspecific binding to endogenous biomolecules^7, 8^. Strategies to reduce false-positive signals include conjugating MBs to macromolecules to inhibit nuclear entry^8–10^, and synthesizing MBs with chemically-modified backbones to increase biostability^11–14^. Based on the latter approach, we recently showed that the 2Me/PS_LOOP_ architecture, which incorporates phosphorothioate (PS) linkages throughout the loop domain of a 2Me MB backbone, enables accurate imaging of single mRNAs harboring 32 tandem repeats of a target sequence with minimal nonspecific signal^11^.

Because RNAs engineered with large sequence insertions could potentially exhibit altered functions or activities, in this study we investigated the minimal target engineering necessary for MB-based imaging of single RNAs using conventional widefield fluorescence microscopy. Using 2Me/PS_LOOP_ MBs, we demonstrated that an RNA engineered with 8 target repeats could be detected in cells with high accuracy, while showing reduced interference with normal RNA trafficking as compared with RNAs engineered with larger target inserts. We further demonstrated the first report of MB-based imaging of dynamics and localization of long noncoding RNAs (lncRNAs) in living cells at the single-molecule level. We anticipate the MB-based approach for imaging single RNAs with minimal target engineering developed in this study can be useful in furthering our understanding of the role of RNA trafficking and localization in health and disease.

## Materials and Methods

### Plasmid Construction

pGEM-1x, pGEM-2x, pGEM-4x, pGEM-8x, pGEM-16x, which encode transcripts containing 1, 2, 4, 8, and 16 tandem repeats of the 50-base sequence 5′-CAGGAGTTGTGTTTGTGGACGAAGAGCACCAGCCAGCTGATCGACCTCGA-3′ (the underlined sequence is the unique MB target site) were kind gifts of Dr. Sanjay Tyagi, Rutgers University, NJ, USA. The derivative constructs, pEGFP-N1-1x, pEGFP-N1-2x, pEGFP-N1-4x, pEGFP-N1-8x and pEGFP-N1-16x, were generated using the same procedure to create pEGFP-N1-32x as described previously^11^. In brief, they were constructed by inserting the EcoRI-BamHI digested fragments of the parental pGEM constructs into pEGFP-N1 (Clontech). The pBFP-N1-8x construct was generated by inserting the EcoRI-BamHI digested fragment of pEGFP-N1-8x into pmTagBFP2-N1 (a kind gift of Dr. Prabuddha Sengupta, Janelia Research Campus, US). The pNEAT1-8x construct was created by PCR amplification of the MS2-NEAT1 plasmid (a kind gift of Dr. Yangming Wang, Peking University, CN) with forward primer 5′-ACCTGCGGATCCGGAGTTAGCGACAGGGAGGGATGCGCGC-3′ and reverse primer 5′-ACTGCTGCGGCCGCTTGTGCTGTAAAGGGGAAGAAA-3′. The PCR product was then inserted into pEGFP-N1-8x backbone digested with BamHI and NotI to excise EGFP. The pHOTAIR-8x construct was created by PCR amplification of cDNA from HEK293 cells with forward primer 5′-AGAACCGGTGACTCGCCTGTGCTCTGGAGCTTGATCCGA-3′ and reverse primer 5′-ATAAGAATGCGGCCGCTTTTTTTTTTGAAAATGCATCCAGATATTA-3′. The PCR product was then inserted into the AgeI- and NotI-digested pEGFP-N1-8x. The EYFP-PSP1α construct was a kind gift of Dr. Archa H. Fox, University of Western Australia, AUS and Dr. Angus I. Lamond, University of Dundee, UK.

### Synthesis of MBs

The 2Me/PS_LOOP_ anti-repeat MB (anti-repeat MB) is labeled with an ATTO647NN reporter dye at the 5′ end and an Iowa Black RQ-Sp quencher at the 3′ end and has the sequence: 5′-mCmUmUmCmG*mU*mC*mC*mA*mC*mA*mA*mA*mC*mA*mC*mA*mA*mC*mU*mC*mC*mU*mGmAmAmG-3′ (m represents 2′-O-methyl RNA modification; * represents PS linkage modification). The control MB is labeled with an ATTO647NN reporter dye at the 5′end and an Iowa Black RQ quencher at the 3′end and has the sequence: 5′-mCmUmCmAmG*mC*mG*mU*mA*mA*mG*mU*mG*mA*mU*mG*mU*mC*mG*mU*mG*mA*mCmUmGmAmG-3′. The MB sequences are designed to avoid hybridization with endogenous RNAs in mammalian cells. All MBs were synthesized by Integrated DNA Technologies (Coralville, IA, USA).

### Cell culture and stable cell line construction

HEK293, NIH3T3, COS7, HeLa cells (American Type Culture Collection) and HeLa cells stably expressing the tandem repeat constructs were cultured in Dulbecco’s Modified Eagle’s Medium without phenol red (DMEM, Mediatech), supplemented with 10% (vol/vol) FBS (PAN Biotech), 1xGlutaMAX (Thermo Fisher) at 37 °C, 5% (vol/vol) CO_2_, and 90% relative humidity.

HeLa cell lines stably expressing pEGFP-N1 (0x), pEGFP-N1-1x, pEGFP-N1-2x, pEGFP-N1-4x, pEGFP-N1-8x, and pEGFP-N1-16x, denoted as HeLa-N1-0x, HeLa-N1-1x, HeLa-N1-2x, HeLa-N1-4x, HeLa-N1-8x, and HeLa-N1-16x, respectively, were generated according to procedures previously used to generate HeLa-N1-32x cells stably expressing pEGFP-N1-32x^11^. In brief, HeLa cells were transfected with plasmids expressing EGFP mRNA harboring various numbers of tandem repeats of the MB target sequence using Fugene HD (Promega). 24 h following the transfection, cells were cultured in media containing 0.8–3 mg/mL geneticin for 3 weeks. Single colonies were isolated and maintained in the absence of any antibiotics. The presence of the engineered repeats was confirmed by smFISH against the MB target sequence. Clones that showed individual RNA punctae easily distinguishable from one another were used for further studies. The same procedure was used to create HeLa cells stably expressing pNEAT1-8x (HeLa-NEAT1-8x), pHOTAIR-8x (HeLa-HOTAIR-8x) and pBFP-N1-8x (HeLa-BFP-N1-8x).

### Cellular delivery of MBs

#### Microporation

Microporation was used in this study as the delivery method for MBs because it enables efficient delivery of MBs into a large number of cells with high viability, as shown previously^15^. In brief, after cells were trypsinized, washed with 1xPBS and pelleted, they were then resuspended in 11 µL resuspension buffer R (Thermo Fisher) so that the final cell concentration was 5,000 cells per µL and the MB concentration was 1 or 5 µM. A Neon transfection system (Thermo Fisher) was used with parameters set at 1005 V, 35 ms pulse width and 2 pulses total for HeLa, 1150 V with a 20 ms pulse width and 2 pulses total for HEK293 cells, 1050 V with a 30 ms pulse width and 2 pulses total for COS7 cells and 1200 V with a 20 ms pulse width and 3 pulses total for NIH3T3 cells. Following microporation and three washes in culture medium to remove free probes, cells were seeded on fibronectin-coated 8-well Lab-Tek Chambered Coverglass (Nunc, Thermo Fisher) for imaging at the indicated time points.

#### Microinjection

All microinjection experiments were performed using a Femtojet and Injectman NI2 (Eppendorf) microinjection system fitted with Femtotips I (Eppendorf). Cells were incubated in DMEM with no phenol red, supplemented with 10% FBS, in glass bottom dishes (Mattek) for all injection experiments.

In addition to microporation and microinjection, we anticipate that other cellular delivery methods previously used in MB research, including lipofectamine, nanoparticles, TAT-peptide, and streptolysin-O^13, 16–22^, should be capable of delivering the MBs used in this study as well.

### Fluorescence microscopy

All microscopy experiments were performed on an Olympus IX 83 motorized inverted fluorescence microscope equipped with a 100x UPlanSApo 1.4NA or a 60x PlanApo N 1.42NA objective lens, back-illuminated EMCCD camera (Andor), Sutter excitation and emission filter wheels and an MT-20E excitation source (Olympus), controlled by CellSens Dimension software. Images were acquired using the Olympus MT20 filter set for DAPI, EGFP and TAMRA and a Chroma filter set for Cy5 (ET620/60x, ET700/75 m, T660lpxr, Chroma). Three-dimensional image stacks were acquired with 0.25 µm increments in the z-direction. All images were analyzed with Fiji^23^ or custom-written MATLAB (Version R2014b 64-bit, MathWorks) programs.

### Single-molecule fluorescence *in situ* hybridization

Single-molecule fluorescence *in situ* hybridization was performed as previously described with modifications^24^. In brief, cells previously microporated with MBs cultured in 8-well chambered coverglass (50–70% confluency) were fixed in 1xPBS solution containing 4% (wt/vol) paraformaldehyde for 30 min at room temperature, washed with 1xPBS, and permeabilized at 4 °C in 70% (vol/vol) ethanol overnight. On the following day, cells were washed thrice with wash buffer [2xSSC, 10% (vol/vol) formamide] and then incubated in hybridization buffer [10% (wt/vol) dextran sulfate, 2xSSC, 10% (vol/vol) formamide] containing a pool of singly-TAMRA-labeled oligonucleotides complementary to different regions of the EGFP coding sequence^11^, the BFP coding sequence (Table S1), or the human HOTAIR sequence (Table S2) (total probe concentration = 250 nM) for 24 h at 37 °C in a humidified chamber. Slides were washed with wash buffer followed by 2xSSC to remove any unbound probes and incubated in 1xPBS prior to imaging.

### Preparation of synthetic RNA transcripts and their hybrids with molecular beacons

To produce synthetic RNAs possessing 8 tandem repeats of the MB target sequence, pGEM-8x plasmid was linearized with NotI and used as a template for *in vitro* transcription by T7 RNA polymerase using the RiboMAX^TM^ large-scale RNA production system per the manufacturer’s protocol. The resulting RNA was purified using lithium chloride precipitation followed by ethanol precipitation as previously described^8^. The concentration of the resulting RNA transcripts was determined spectrophotometrically. Hybrids were formed by incubating MBs and transcripts at 50:1 molar ratio in 1xPBS for 60 min. The mixture was then microinjected or microporated into HeLa-N1-0x cells at the final concentration of 5 µM MBs and 0.1 µM transcripts following the microporation protocol described above.

### Identification of single RNA transcripts

Single RNA transcripts in HeLa cells stably expressing different numbers of tandem repeats of the MB target sequence were identified as described previously^25^. In brief, rolling-ball background subtraction (background = 2) was used on all 3D images, followed by identification of particles using the 3D Laplacian of Gaussian plug-in available for Fiji^23^. After low-intensity spots were removed, a region of interest (ROI) was drawn around individual cells and applied to the filtered stack. The Find Stack Maxima macro plug-in (Exclude Edge Maxima; Noise Tolerance = 10) was used to identify all local maxima in each slice of the z-stack. To identify which 2D local maxima were 3D local maxima, a custom MATLAB program compared the intensity of each local maximum in each slice with the intensity of the neighboring pixels in the current slice and the two adjacent slices (9 pixels in the slice above, 8 surrounding pixels in the same slice, and 9 pixels in the slice below). Each 3D maximum was considered to be an individual RNA.

### RNA colocalization

After determining the 3D coordinates of RNA transcripts in smFISH and MB images using the methods described above, a custom MATLAB program was used to identify the extent of colocalization in three-dimensions as previously described^25^. In brief, an MB 3D local maximum was considered to be a colocalization event if an smFISH 3D local maximum was found within a 3 × 3 × 3 voxel cube centered around the MB maximum. The colocalization percentage was calculated by dividing the number of colocalization events by the total number of smFISH local maxima.

### Single-particle tracking analysis

All images were acquired at 100 ms per frame to identify and localize single engineered RNA transcripts using the TrackMate plugin for Fiji, as shown previously^11^. In brief, Laplacian of Gaussian followed by Differences of Gaussian filters were used to identify individual peaks and their coordinates^26^. Peaks that belong to the same track were determined by simple Linear Assignment Problem tracker (linking max distance = 1 μm; gap-closing max distance = 2 μm; gap-closing max frame gap = 4). The assigned tracks were then imported into @msdanalyzer written in MATLAB^27^. Tracks containing at least 15 time lags (Δτ) were selected for calculating the Mean Square Displacement (MSD). For simplicity, the 2-dimensional diffusion coefficient D_eff_ was obtained from a linear fitting of MSD vs. Δτ, using the first 25% of total time lags, with a minimum fitting threshold of R^2^ > 0.9. The minimum D_eff_ threshold for mobile fractions was set at 0.0006 μm^2^/s, using D_eff_ calculated for Tetraspek beads (Thermo Fisher) immobilized on a coverslip as a control for xy-drift.

### Quantification of single particle intensity

To quantify fluorescence intensity of each particle, all images were acquired for 100 ms and analyzed in Fiji. In brief, particulate objects were first enhanced by applying rolling-ball background subtraction (background = 2) to all images. Appropriate thresholds were then set to filter out background noise. The total integrated intensity of each particle was analyzed using the “analyze particles” tool, with the size of particles set to be at least 3 × 3 pixels.

### Data analysis

All experiments were repeated at least three times and statistics were performed using t-test or one-way ANOVA with post hoc testing of pairwise comparisons using Fisher’s protected least significant difference unless otherwise stated. Kolmogorov-Smirnov test was used to further analyze distributions in Figs S9, S10 and S11. Significant difference was set at the p < 0.05 level.

## Results and Discussion

### Detection of single RNA transcripts engineered with different numbers of target repeats

**Figure 1:**
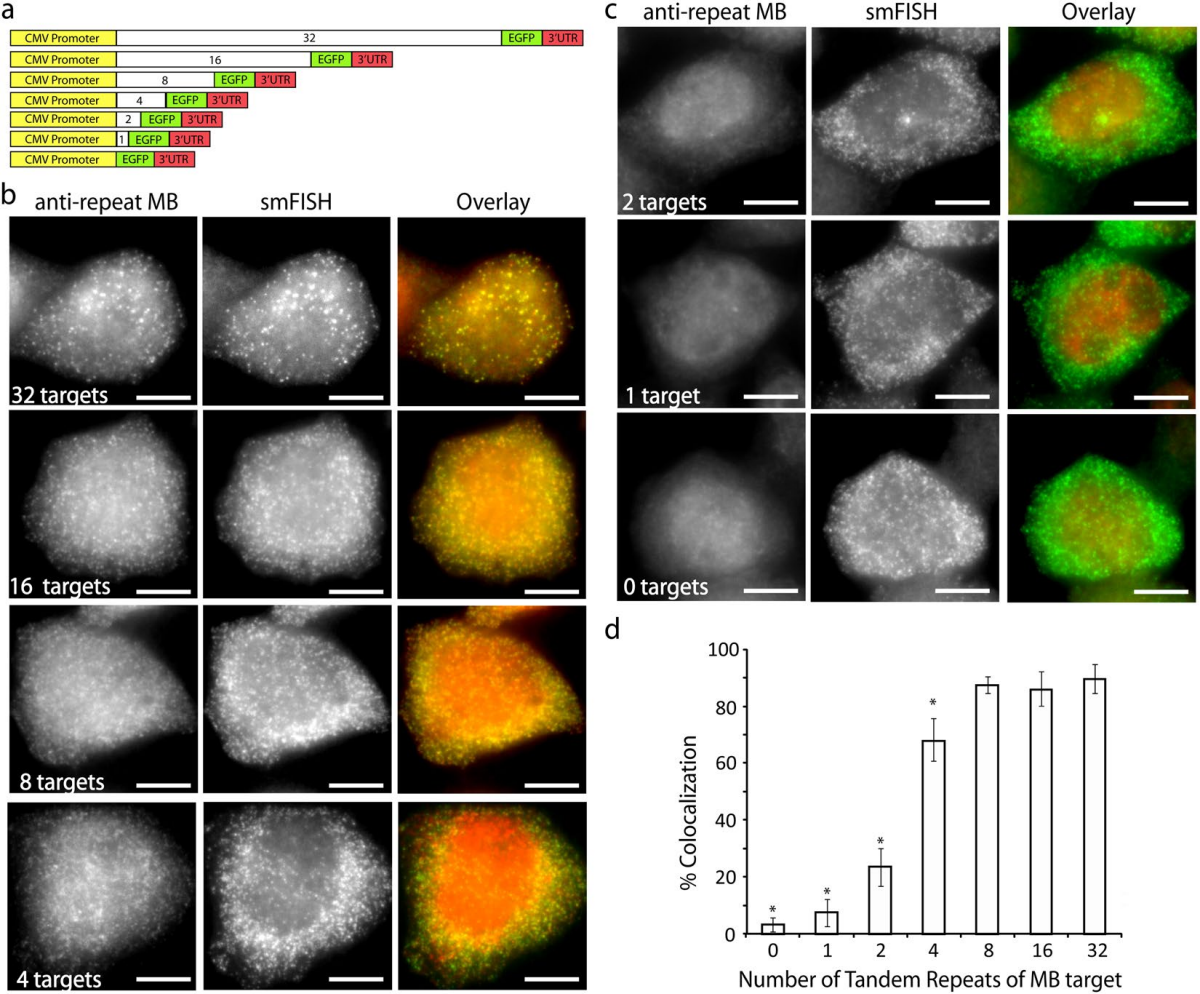
Detection of single RNA transcripts harboring different tandem repeats of MB binding sites using anti-repeat MBs and smFISH. (**a**) Scheme of the 7 constructs expressing EGFP with tandem repeat sequences inserted upstream of the EGFP gene. The MB targets and the EGFP mRNA are transcribed as one molecule. After microporation of HeLa cells expressing EGFP mRNA harboring different numbers of MB targets with 5 µM anti-repeat MBs, the cells were fixed and permeabilized and smFISH was performed to assess the accuracy of MBs for detecting single RNA transcripts. Representative maximum intensity projection images of anti-repeat MBs (ATTO647N-labeled) and EGFP smFISH (TAMRA-labeled) in HeLa cells expressing EGFP engineered with (**b**) 32, 16, 8, or 4, and (**c**) 2, 1, or 0 tandem repeats of MB target sequence at 8 h are shown. (Scale bar, 10 µm) (**d**) Accuracy of anti-repeat MBs for the detection of single RNA transcripts engineered with different numbers of MB binding sites. A custom Matlab program was written to analyze the percentage of MB signals that colocalized with smFISH signals. Data represent mean ± SD of at least 10 cells. *Represents significant difference from the 32-repeats construct.

Previously, we showed that 2Me/PS_LOOP_ MBs complementary to a repeat target sequence (anti-repeat MBs, Fig. S1) can detect single mRNAs engineered with 32 tandem repeats of the MB target sequence with ~90% accuracy^11^. To determine whether anti-repeat MBs can be used to detect RNAs harboring fewer target sequences, we created HeLa cell lines stably expressing engineered RNA transcripts that harbor 0, 1, 2, 4, 8, 16 or 32 tandem repeats of an MB target sequence upstream of the EGFP coding sequence (see Materials and Methods) (Fig. 1a). Since both the tandem repeats and the EGFP coding sequence are transcribed as one RNA molecule, we hypothesize that if sufficient numbers of MBs hybridize to the engineered transcripts, the resulting MB signals should appear as discrete bright spots that colocalize with smFISH signals that identify single RNA transcripts.

Consistent with our previous findings, after HeLa-N1-32x cells microporated with anti-repeat MBs were processed by smFISH against EGFP, discrete bright spots were readily detected in both the nucleus and the cytoplasm, as shown in the maximum intensity projection images acquired in the respective fluorescence channels (Fig. 1b)^11^. Similarly, discrete MB and smFISH signals were visible in the nucleus and the cytoplasm of HeLa-N1-16x, -8x, and -4x cells. In cells expressing EGFP transcripts harboring fewer than 4 repeats (Fig. 1c), the majority of the MB signals were observed in the nucleus. In these cells, MB signals appeared to be more diffusely distributed in both the nucleus and the cytoplasm, whereas the smFISH signals, identifying single mRNAs, still exhibited the expected punctate staining pattern in the nucleus and the cytoplasm. The nuclear localization and diffuse signal of the MBs seen in HeLa-N1-2x, HeLa-N1-1x and HeLa-N1-0x suggests that an insufficient number of target repeats bound to MBs, failing to produce an identifiable fluorescence signal. This also suggests that unbound MBs may be more susceptible to nuclear sequestration and subsequent nonspecific fluorescence. In all cells, EGFP fluorescence confirmed presence of active EGFP mRNA (Fig. S2), in agreement with our previous findings that binding of anti-repeat MBs to pEGFP-N1-32x mRNA does not affect translation^11^.

Quantification of colocalization in 3-dimensions (see Materials and Methods) revealed that in HeLa-N1-32x cells, 90% ± 5% of the MB spots colocalized with smFISH spots at 8 h post-microporation (Fig. 1d), in agreement with our previous analysis^11^. The extent of colocalization was 85% ± 7% in HeLa-N1-16x cells and 88% ± 3% in HeLa-N1-8x cells. There was no significant difference in colocalization between the HeLa-N1-32x, -16x and -8x cells. In cells expressing EGFP mRNA harboring fewer than 8 repeats, a reduction in colocalization was observed, with the extent of colocalization decreasing with decreasing numbers of MB target repeats. Specifically, the extent of colocalization was 68% ± 8% in HeLa-N1-4x cells, 24% ± 7% in HeLa-N1-2x cells, 7% ± 5% in HeLa-N1-1x cells and 3% ± 3% in HeLa-N1-0x cells. The decrease in colocalization is assumed to be due to loss of MB signals owing to insufficient detection sensitivity, since smFISH spots were readily detectible in these cells. Consistent with this, lower colocalization was detected in cells with each target repeat construct when MBs were microporated at lower concentration (1 µM) (Fig. S3). Furthermore, the extent of colocalization between MB and smFISH signals was 87% ± 5% in HEK293 cells (Fig. S4) and 93% ± 4% in COS7 cells (Fig. S5) expressing pEGFP-N1-8x, and 85% ± 6% when a different transcript that contains 8 tandem target repeats upstream of the pTagBFP transcript (pBFP-N1-8x) was expressed in HeLa cells (Fig. S6). Finally, control MBs designed to not bind the repeat sequence showed negligible colocalization with smFISH signals in HeLa-N1-8x cells (1% ± 1%) and HeLa-N1-32x cells (2% ± 3%), confirming the specificity of anti-repeat MBs in detecting the engineered repeats (Figs S7 and S8). Overall, these data indicate that MBs can detect single engineered RNAs containing as few as 8 repeats of a target sequence, and render the RNAs sufficiently fluorescent for imaging by conventional widefield fluorescence microscopy with nearly 90% accuracy. The inability to achieve 100% detection accuracy may reflect false-positives resulting from a low-frequency tendency of the 2Me/PS_LOOP_ MBs to open nonspecifically in cells^11^ and/or detection of tandem repeats that are potentially truncated from the EGFP coding sequence. It should be noted that in addition to 2Me/PS_LOOP_ MBs, other biosensors with high reported biostability, including MBs synthesized with backbones containing locked nucleic acids^14^ or modified with structures that minimize nuclear entry^8–10^, are likely also capable of accurately detecting minimally engineered RNAs. The need to target only 8 repeats to achieve high accuracy in single-molecule RNA detection also suggests the possibility of accurately imaging endogenous RNAs in their native cellular contexts at the single molecule level within reasonable cost and design feasibility constraints by using eight different MB sequences.

### Single-particle tracking analysis reveals that RNA transcripts harboring fewer numbers of repeats exhibit greater intracellular mobility

One concern when engineering RNAs with large sequence insertions is the potential to alter RNA trafficking, causing changes in their functions and activities. Based on the above finding that anti-repeat MBs can detect both pEGFP-N1-32x and pEGFP-N1-8x transcripts with ~90% accuracy, we next performed single-particle tracking analysis to determine whether the pEGFP-N1-32x and pEGFP-N1-8x transcripts, which are ~2300 nucleotides (nt) long and ~1100 nt long, respectively, exhibit a difference in intracellular mobility. To investigate this, HeLa-N1-32x and HeLa-N1-8x cells were microporated with anti-repeat MBs and time-lapse images were acquired at 8 h post-microporation (Movies S1 and S2). Individual bright spots, indicating single pEGFP-N1-32x or pEGFP-N1-8x transcripts, could be readily observed in both the nucleus and the cytoplasm in living cells. By contrast, MBs in HeLa-N1-1x and HeLa-N1-2x cells exhibited few or no fluorescent spots (See Movies S3 and S4) whereas the MBs in HeLa-N1-4x cells appear to be less distinguishable from background (Movie S5), consistent with the colocalization analysis (See Fig. 1).

**Figure 2:**
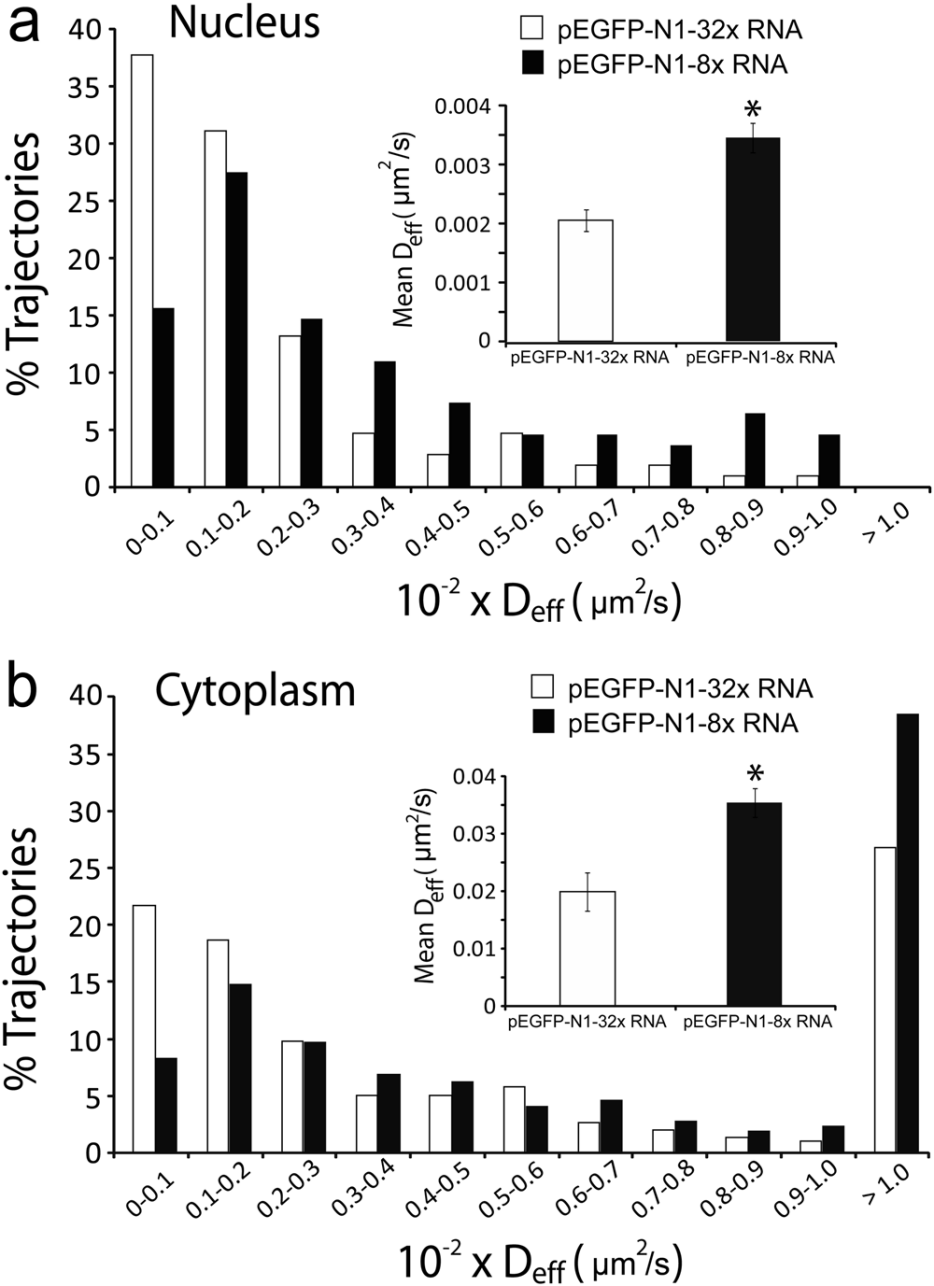
Diffusion kinetics of single EGFP mRNA transcripts harboring 32 or 8 MB target sequence repeats in the nucleus and the cytoplasm. HeLa-N1-32x and HeLa-N1-8x cells were microporated with 5 µM anti-repeat MBs, and time-lapse images were acquired 8 h after microporation (See Movies S1 and S2). Single particle tracking analysis was performed to determine the diffusion coefficient of individual RNAs as described in Materials and Methods. (**a**) The distribution of diffusion coefficients of single mobile pEGFP-N1-32x RNAs in the nucleus (n = 106 tracks) and in the cytoplasm (n = 295 tracks) analyzed from at least 23 cells. (**b**) The distribution of diffusion coefficients of single mobile pEGFP-N1-8x RNAs in the nucleus (n = 109 tracks) and the cytoplasm (n = 1067 tracks) analyzed from at least 33 cells. *Inset* shows the mean ± SE diffusion coefficients.

Single-particle tracking analysis revealed that nearly 65% and 35% of the pEGFP-N1-32x RNAs in the nucleus and the cytoplasm, respectively, were immobile. The mean diffusion of mobile pEGFP-N1-32x RNAs was 0.0020 ± 0.0002 µm^2^/s in the nucleus and 0.0199 ± 0.0033 µm^2^/s in the cytoplasm (Fig. 2a,b), similar to our previous analysis^11^. By contrast, only 35% and 12% of the pEGFP-N1-8x RNAs in the nucleus and the cytoplasm, respectively, were immobile. The mean diffusion of mobile pEGFP-N1-8x RNAs was 0.0035 ± 0.0003 µm^2^/s in the nucleus and 0.0355 ± 0.0025 µm^2^/s in the cytoplasm (Fig. 2a,b). Both transcripts, despite the difference in length, moved slower in the nucleus than in the cytoplasm, consistent with the idea that the nucleus is more viscous than the cytoplasm^28^. Furthermore, pEGFP-N1-8x RNAs moved faster than pEGFP-N1-32x RNAs in both the nucleus and the cytoplasm, indicating that large sequence insertions may significantly impede the intracellular movement of target RNAs. Additionally, the particle intensity profile of MBs in HeLa-N1-8x cells is similar to that of the *in vitro* prepared MB:8x transcript hybrid in HeLa-N1-0x cells, suggesting that the majority of the detected particles in HeLa-N1-8x cells are single RNA transcripts (Figs S9 and S10). Together, these findings demonstrate that shortening the engineered insertion can have reduced impact on RNA dynamics at the single molecule level.

### Live-cell imaging of single lncRNA transcripts

Long noncoding RNAs (lncRNAs), a class of transcripts greater than 200 nucleotides long, are emerging as crucial regulators of diverse biological functions at epigenetic, transcriptional and post-transcriptional levels^29–31^. To date, single-molecule dynamics of lncRNAs in living cells have been predominantly studied with the MS2-FP system^32, 33^. Our finding that mRNAs engineered with eight tandem target repeats could be imaged with ~90% accuracy and with reduced impact on RNA dynamics led us to investigate whether the same approach can be used to study single lncRNAs. We tested this idea with human Nuclear Enriched Abundant Transcript 1 (NEAT1) and (HOX Transcript Antisense RNA) HOTAIR, lncRNAs that have emerging roles in the regulation of cellular behavior and disease progression^34–37^.

**Figure 3:**
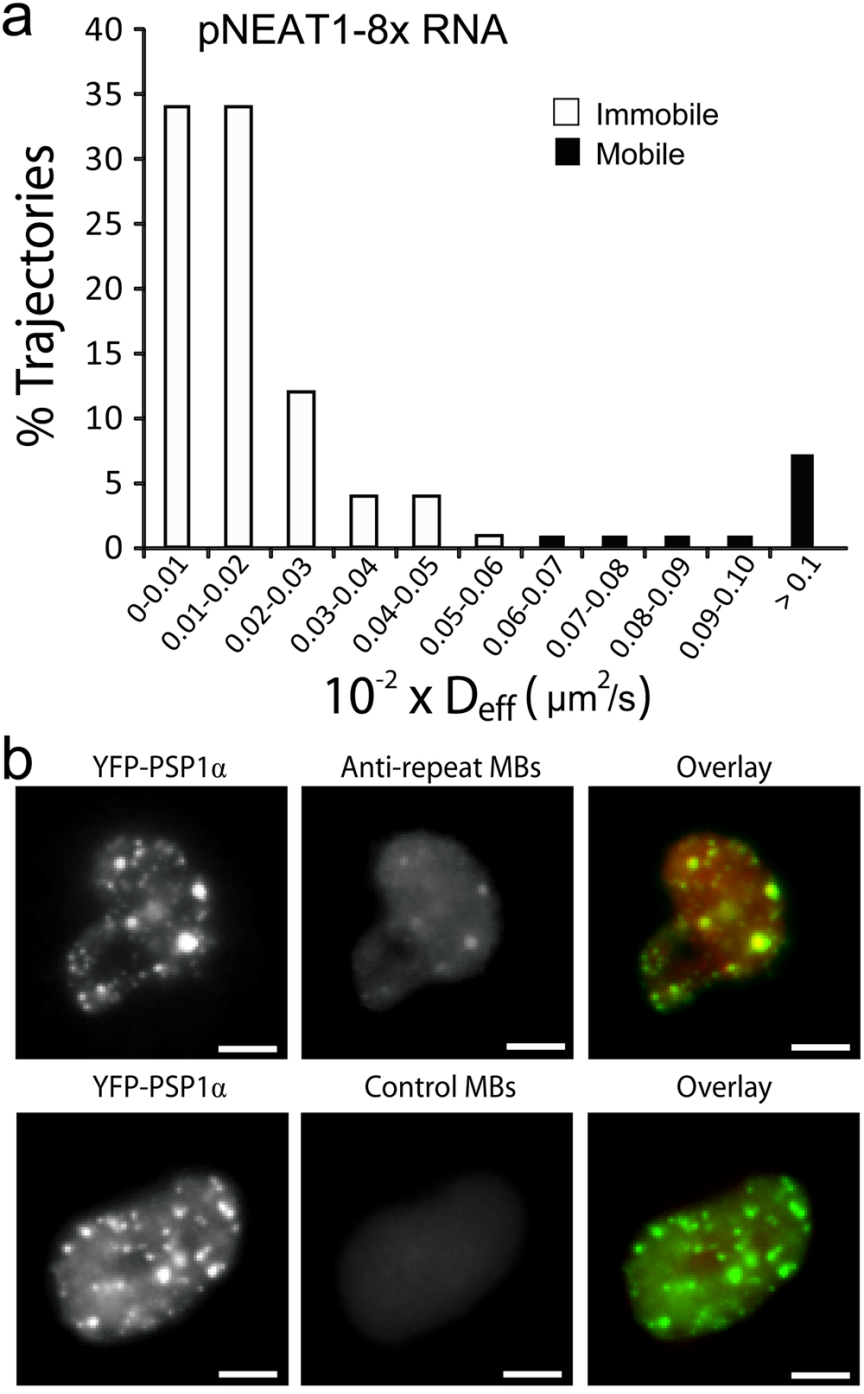
MB-based imaging of single NEAT1 transcripts in the nucleus. (**a**) Diffusion kinetics of single engineered NEAT1 lncRNA transcripts in the nucleus. HeLa-NEAT1-8x cells were microporated with 5 µM anti-repeat MBs, and time-lapse images were acquired 8 h after microporation (See Movie S6). Single particle tracking analysis was performed to determine the diffusion coefficient of individual RNAs as described in Materials and Methods. The distribution of diffusion coefficients of single mobile pNEAT1-8x RNAs in the nucleus (n = 200 tracks) were analyzed from 25 cells. Data representing immobile tracks (D_eff_ < 0.0006 µm^2^/s as defined by immobilized Tetraspek beads) are highlighted in white. Data representing mobile tracks are highlighted in black. 89% of the detected engineered NEAT1 transcripts are immobile. (**b**) Live-cell detection of NEAT1 colocalization with PSP1α proteins. 5 µM of anti-repeat MBs or control MBs were injected into HeLa-NEAT1-8x cells transfected with pEYFP-PSP1α constructs. Representative fluorescent images acquired within a few minutes post-injection are shown. (Scale bar, 10 µm).

NEAT1 is a ~3700 nt lncRNA that predominantly resides in the nucleus and is essential for the formation of paraspeckles, which are ribonucleoprotein complexes with reported regulatory function in various biological and pathological processes^34, 35^. Consistent with its nuclear localization property, time-lapse images, acquired at 8 h following microporation of anti-repeat MBs into HeLa cells stably expressing pNEAT1-8x, revealed individual bright spots indicating single pNEAT1-8x transcripts (~4.1 kb in length) localized predominantly in the nucleus (Movie S6). Single-particle tracking analysis showed that 89% of the detected particles (n = 200) within the nucleus were immobile (D_eff_ < 0.0006 µm^2^/s), with the remaining mobile particles moving only at 0.0019 µm^2^/s ± 0.0004 µm^2^/s (Fig. 3a). The lack of movement, as compared with EGFP mRNAs, likely reflects the binding of NEAT1 lncRNAs to nuclear proteins in the interchromatin space as previously characterized^34, 35^.

Previous studies have reported that NEAT1 associates with paraspeckle protein 1 (PSP1) to form paraspeckles^34, 35^. To investigate whether the observed immobile properties of pNEAT1-8x RNAs arose from NEAT1/PSP1 interaction, plasmid constructs encoding EYFP-PSP1α were transiently expressed in HeLa-NEAT1-8x cells. Fluorescence microscopy images revealed that EYFP-PSP1α exhibited a punctate fluorescence signal (Fig. 3b), resembling formation of paraspeckles as shown previously^35^. Immediately following microinjection of the anti-repeat MBs into cells expressing EYFP-PSP1α, a punctate MB signal was also observed in the nucleus. The MB signal puncta were not observed after microinjection of control MBs into HeLa-NEAT1-8x cells expressing EYFP-PSP1α, confirming the specificity of anti-repeat MBs for pNEAT1-8x transcripts. Overall, these studies showed that NEAT1 transcripts are highly immobile in living cells and suggest that MB-based imaging does not impact the activities of the targeted lncRNAs.

**Figure 4:**
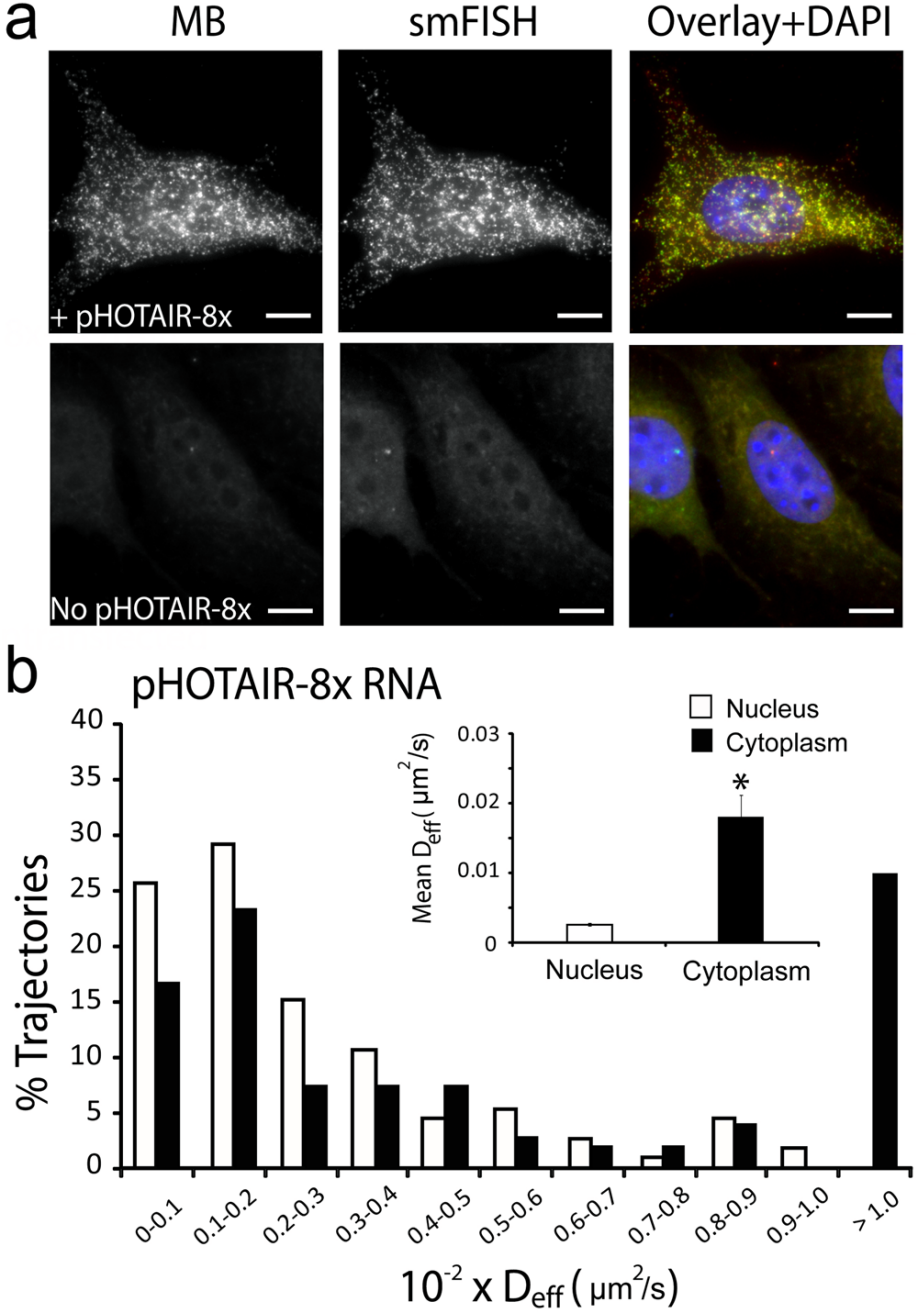
MB-based imaging of single HOTAIR transcripts in the nucleus and the cytoplasm. (**a**) Images of anti-repeat MBs for the detection of single pHOTAIR-8x transcripts. Following microporation of NIH3T3 cells with or without pHOTAIR-8x with 5 µM of anti-repeat MBs, the cells were fixed and permeabilized and smFISH was performed to assess the accuracy of MBs for detecting single RNA transcripts. Representative maximum intensity projection images of MB and smFISH signals are shown. Note that in NIH3T3 cells not transfected with pHOTAIR-8x, lack of smFISH signal shows the specificity of the smFISH probes for the human HOTAIR transcript used. (Scale bar, 10 µm) (**b**) Diffusion kinetics of single pHOTAIR-8x lncRNA transcripts in HeLa cells. HeLa-HOTAIR-8x cells were microporated with 5 µM anti-repeat MBs, and time-lapse images were acquired 8 h after microporation. Single particle tracking analysis was performed to determine the diffusion coefficient of individual RNAs as described in Materials and Methods. The distribution of diffusion coefficients of single mobile pHOTAIR-8x RNAs in the nucleus (n = 113 tracks) and in the cytoplasm (n = 255 tracks) analyzed from 30 cells. *Inset* shows the mean ± SE diffusion coefficients.

HOTAIR is a ~2.200 nt lncRNA that has been shown to regulate cellular processes at both the epigenetic and post-translational levels^36, 37^. Consistent with these reported functions, following microporation of the anti-repeat MBs into HeLa cells transfected with pHOTAIR-8x transcripts (~2.6 kb) (HeLa-HOTAIR-8x), bright spots, indicative of single pHOTAIR-8x transcripts, were observed in both the nucleus and in the cytoplasm (Movie S7). Evidence that the MBs can specifically detect pHOTAIR-8x transcripts came from MB and smFISH studies in NIH3T3 cells, in which the endogenous murine HOTAIR transcripts cannot be detected by smFISH probes targeting the human HOTAIR (Fig. 4a). In NIH3T3 cells transfected with plasmid encoding the pHOTAIR-8x transcripts, MB signal colocalized extensively with smFISH signals (94% ± 3%), suggesting that MBs could accurately monitor the intracellular dynamics and localization of pHOTAIR-8x transcripts in living cells.

Single-particle tracking analysis showed that 41% of the detected pHOTAIR-8x transcripts (n = 193) in the nucleus were immobile, with the remaining mobile fraction moving at 0.0026 ± 0.0002 µm^2^/s (Fig. 4b). In the cytoplasm, 24% of the detected pHOTAIR-8x transcripts (n = 337) were immobile, with the remaining mobile molecules moving at 0.0181 ± 0.0030 µm^2^/s. The slow diffusion in the nucleus as compared with the cytoplasm is consistent with the idea that nucleus is a more viscous environment than the cytoplasm^28^. Additionally, the particle intensity profile of MBs in HeLa-HOTAIR-8x cells is in good agreement with that of the MB:8x transcript hybrid prepared *in vitro* in HeLa-N1-0x cells, suggesting the majority of the detected particles in HeLa-HOTAIR-8x cells are single pHOTAIR-8x transcripts (Figs S9 and S11). Compared with the pEGFP-8x, pHOTAIR-8x exhibits slower mobility in both the nucleus and the cytoplasm, as expected since pHOTAIR-8x transcripts are larger than pEGFP-8x transcripts. Interestingly, the pHOTAIR-8x transcripts have similar diffusion properties as those of the pEGFP-32x transcripts in both the nucleus and the cytoplasm. As pEGFP-32x and pHOTAIR-8x transcripts belong to different classes of RNAs and have very different functions, but are similar in size, the similarity in diffusion properties between the two transcripts suggests that diffusion of mRNAs and lncRNAs within HeLa cells is largely dependent on the transcript size and independent of RNA class. Presumably, both RNAs interact with respective partner proteins to form ribonucleoprotein complexes that exhibit similar diffusion characteristics. It is also possible that both transcripts are transported via the same RNA transport machineries.

To our knowledge, this is the first study that visualizes NEAT1 and HOTAIR lncRNAs in living cells at the single molecule level. With potential for minimized impact on RNA biology, we envision the MB-based imaging approach can be applied to study other RNA molecules, elucidating their functions with previously unattainable detail and resolution.

## Conclusion

With the growing interest in deciphering the role of RNAs in health and disease, it has become essential to be able to visualize single RNA transcripts in living cells with high spatial and temporal resolutions. Using MBs that elicit a marginal level of nonspecific signals (2Me/PS_LOOP_ MBs), we showed that single RNA transcripts engineered with as few as 8 tandem repeats of an MB target sequence can be detected at the single-molecule level with high accuracy under standard widefield fluorescence microscopy. Shortening the engineered insertion was also shown to reduce the impact on the mobility of intracellular RNAs. Additional studies showed for the first time that MBs can be used to image lncRNAs and monitor their activities at the single-molecule level in living cells.

To date, diffusion coefficients for RNA movement in mammalian cells have been reported to range from 0.0006 to 3.42 µm^2^/s in the nucleus^6, 38–41^ and from 0.011 to 2.24 µm^2^/s in the cytoplasm^6, 38, 40, 42^. This range of measurements can be due to several factors, including differences in the nature and the functions of the detected RNA species, the size of the RNAs, the number and the types of proteins or other RNAs within the same ribonucleoprotein complex, cell types, and methods used for imaging and analysis. Since diffusion coefficient values of the different target transcripts detected in this study fit within the range of the reported values in the literature, we are confident our MB-based approach for live-cell imaging of single RNA transcripts with minimal target engineering is a reliable approach for studying RNA dynamics in living cells.

It should be noted that using MBs to image single RNA transcripts can have several advantages over the widely used MS2-FP approach. For example, the feasibility of incorporating brighter and more photostable fluorophores into MBs could enable imaging of target RNAs over a longer period of time than would be possible when using fluorescent proteins. Additionally, MB fluorophores are quenched in the absence of target RNA, which reduces background signals significantly as compared with the unquenched fluorescent proteins of the MS2 system. Furthermore, the eight tandem MB target repeat sequence (~400 nt) is smaller than an MS2-tag that typically contains 24 tandem repeats of MS2-binding sites (~1,000 nt), so engineered RNAs with the MB tag are expected exhibit less disruption of RNA trafficking, localization and functionality. Finally, MBs (~11 kDa) are smaller than MS2-FP fusion proteins (~44 kDa), and therefore may move more freely in the intracellular environment and may cause less disruption of target RNA activity when bound. This could be crucial in studies of RNAs that localize in the highly crowded environment of the nucleus or are tightly associated with proteins to form ribonucleoprotein complexes. With the increasing need in visualizing RNA molecules with high resolution, accuracy and sensitivity, we envision that the MB-based imaging approach presented here can aid discoveries of new RNA biology.

## Electronic supplementary material

Supplementary Materials

Representative movie of mRNA transcripts in a HeLa-N1-32x cell

Representative movie of mRNA transcripts in a HeLa-N1-8x cell

Representative movie of mRNA transcripts in a HeLa-N1-1x cell

Representative movie of mRNA transcripts in a HeLa-N1-2x cell

Representative movie of mRNA transcripts in a HeLa-N1-4x cell

Representative movie of NEAT1 lncRNA transcripts in a HeLa-NEAT-8x cell

Representative movie of HOTAIR lncRNA transcripts in a HeLa-HOTAIR-8x cell
