# Supplementary material for: A molecular beacon-based approach for live-cell imaging of RNA transcripts with minimal target engineering at the single-molecule level
Source: Sci Rep. 2017 May 8;7:1550. doi: 10.1038/s41598-017-01740-1 (PMC5431543; doi:10.1038/s41598-017-01740-1)
Supplement: Supplementary file 1 — Supplementary Materials [file 41598_2017_1740_MOESM1_ESM.pdf]

A molecular beacon-based approach for live-cell imaging of RNA transcripts with minimal target engineering at the single-molecule level

Mingming Chen[1-3], Zhao Ma[1], Xiaotian Wu[1], Shiqi Mao[1], Yantao Yang[1], Jie Tan[1], Christopher J. Krueger[1, 4] and Antony K. Chen[1,*]

Supplementary Information

SUPPLEMENTARY MATERIALS

Supplementary Figure S1. Scheme of 2Me/PS$_{LOOP}$ MB and backbone structures. The MB, labeled with a reporter dye (red) and a quencher (black), has a backbone composed of 2Me RNAs with a phosphorothioate (PS) loop domain (blue line) and a phosphodiester (PO) stem (black line). The PS bond was synthesized by substituting a sulfur atom for a non-bridiging oxygen in the PO bond.

Supplementary Figure S2. EGFP fluorescence in cells shown in Figures 1B and 1C. (Scale bar, 10 µm)

Supplementary Figure S3. Detection of single RNA transcripts harboring different numbers of MB targets using smFISH and a lower concentration (1 µM) of anti-repeat MBs at 8h post-microporation. The extent of colocalization of MBs to smFISH signals was analyzed from at least 10 cells for each tandem repeat construct. Data represent mean ± SD. * represents significant difference from pEGFP-N1-32x.

Supplementary Figure S4. Detection of single pEGFP-N1-8x mRNAs in HEK293 cells at 8 h post-microporation of anti-repeat MBs. Representative EGFP and maximum intensity projection images of control MBs and EGFP smFISH at 8 h post-microporation are shown. (Scale bar, 10 µm)

Supplementary Figure S5. Detection of single pEGFP-N1-8x mRNAs in COS7 cells at 8 h post-microporation of anti-repeat MBs. Representative EGFP image and maximum intensity projection images of control MBs and EGFP smFISH at 8 h post-microporation are shown. (Scale bar, 10 µm)

Supplementary Figure S6. Detection of single pBFP-N1-8x mRNAs in HeLa cells at 8 h post-microporation of anti-repeat MBs. Representative BFP image and maximum intensity projection images of control MBs and EGFP smFISH at 8 h post-microporation are shown. (Scale bar, 10 µm)

Supplementary Figure S7. Detection of pEGFP-N1-8x RNA transcripts using smFISH and control MBs. After microporation of HeLa-N1-8x cells with 5 µM control MBs, the cells were fixed and smFISH was performed. Representative EGFP fluorescence and maximum intensity projection images of control MBs and EGFP smFISH at 8 h post-microporation are shown. (Scale bar, 10 µm)

Supplementary Figure S8. Detection of pEGFP-N1-32x RNA transcripts using smFISH and control MBs. After microporation of HeLa-N1-32x cells with 5 µM control MBs, the cells were fixed and smFISH was performed. Representative EGFP fluorescence and maximum intensity projection images of control MBs and EGFP smFISH at 8 h post-microporation are shown. (Scale bar, 10 µm)

Supplementary Figure S9. Confirmation that microporated MB:8x transcript hybrids

represent single RNA transcripts. HeLa-N1-0x cells were either microinjected or microporated with a mixture containing prehybridized 5 µM MBs and 0.1 µM 8x synthetic target RNA. The cells were imaged immediately following microinjection or at 8 h post-microporation under the same microscopy settings. Particle intensities were then quantified as described in Materials and Methods. Particle intensity distribution of MB:target hybrids introduced by microinjection (n= 1134 blue bar) and by microporation (n= 607, red bar) are shown. In both cases, particles were analyzed from at least 50 cells. There was no significant difference in the mean (p = 0.232, t-test) and between the two distributions (p = 0.821, Kolmogorov-Smirnov test). Thus, at 8 hours following microporation, the majority of the RNA-MB hybrids could still remain as single hybrids and did not localize to RNA granules.

Supplementary Figure S10. Confirmation that particles represent single EGFP mRNAs. 8 h post-microporation of HeLa-N1-8x cells with 5 µM MBs or HeLa-N1-0x cells with a mixture containing prehybridized 5 µM MBs and 0.1 µM 8x synthetic target RNA, the cells were fixed and imaged under the same microscopy settings. Particle intensities were then quantified as described in Materials and Methods. Particle intensity distributions of MBs in HeLa-N1-8x cells (n = 311, red bar) and of synthetic MB:8x hybrids in HeLa-N1-0x cells (n = 230, blue bar) are shown. In both cases, particles were analyzed from at least 40 cells. There was no significant difference in the mean (p = 0.121, t-test) and between the two distributions (p = 0.861, Kolmogorov-Smirnov test).

Supplementary Figure S11. Confirmation that particles represent single HOTAIR lncRNAs. 8 h post-microporation of HeLa-HOTAIR-8x cells with 5 µM MBs or HeLa-N1-0x cells with a mixture containing prehybridized 5 µM MBs and 0.1 µM 8x synthetic target RNA, the cells were fixed and imaged under the same microscopy settings. Particle intensities were then quantified as described in Materials and Methods. Particle intensity distributions of MBs in HeLa-HOTAIR-8x cells (n = 401, green bar) and of synthetic MB:8x hybrid in HeLa-N1-0x cells (n = 230, blue bar) are shown. In both cases, particles were analyzed from at least 40 cells. There was no significant difference in the mean (p = 0.341, t-test) and between the two distributions (p = 0.987, Kolmogorov-Smirnov test).

Supplementary Table 1. The set of probes against the mTagBFP2 coding sequence used for smFISH imaging of individual engineered constructs. All of the probes were labeled with a TAMRA fluorophore at the 3' end.

Supplementary Table 2. The set of probes against the human HOTAIR coding sequence used for smFISH imaging of individual engineered constructs. All of the probes were labeled with a TAMRA fluorophore at the 3' end.

Supplementary Movie 1. Representative movie of mRNA transcripts in a HeLa-N1-32x cell. The cell is expressing pEGFP-N1-32x RNAs.

Supplementary Movie 2. Representative movie of mRNA transcripts in a HeLa-N1-8x cell. The cell is expressing pEGFP-N1-8x RNAs.

Supplementary Movie 3. Representative movie of mRNA transcripts in a HeLa-N1-1x cell. The cell is expressing pEGFP-N1-1x RNAs.

Supplementary Movie 4. Representative movie of mRNA transcripts in a HeLa-N1-2x cell. The cell is expressing pEGFP-N1-2x RNAs.

Supplementary Movie 5. Representative movie of mRNA transcripts in a HeLa-N1-4x cell. The cell is expressing pEGFP-N1-4x RNAs.

Supplementary Movie 6. Representative movie of NEAT1 lncRNA transcripts in a HeLa-NEAT-8x cell. The cell is expressing pNEAT1-8x RNAs.

Supplementary Movie 7. Representative movie of HOTAIR lncRNA transcripts in a HeLa-HOTAIR-8x cell. The cell is expressing pHOTAIR-8x RNAs.

Chen et. al. Supplementary Figure S1

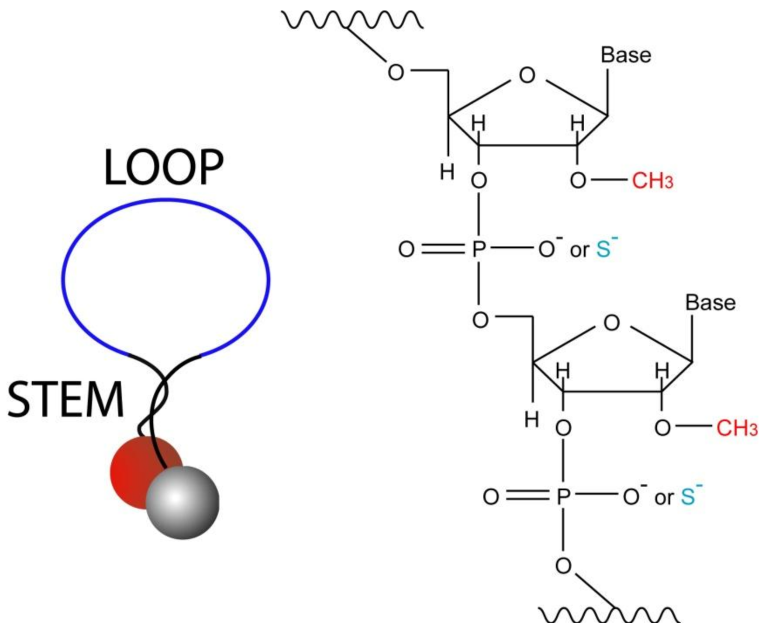

Chen et. al. Supplementary Figure S2

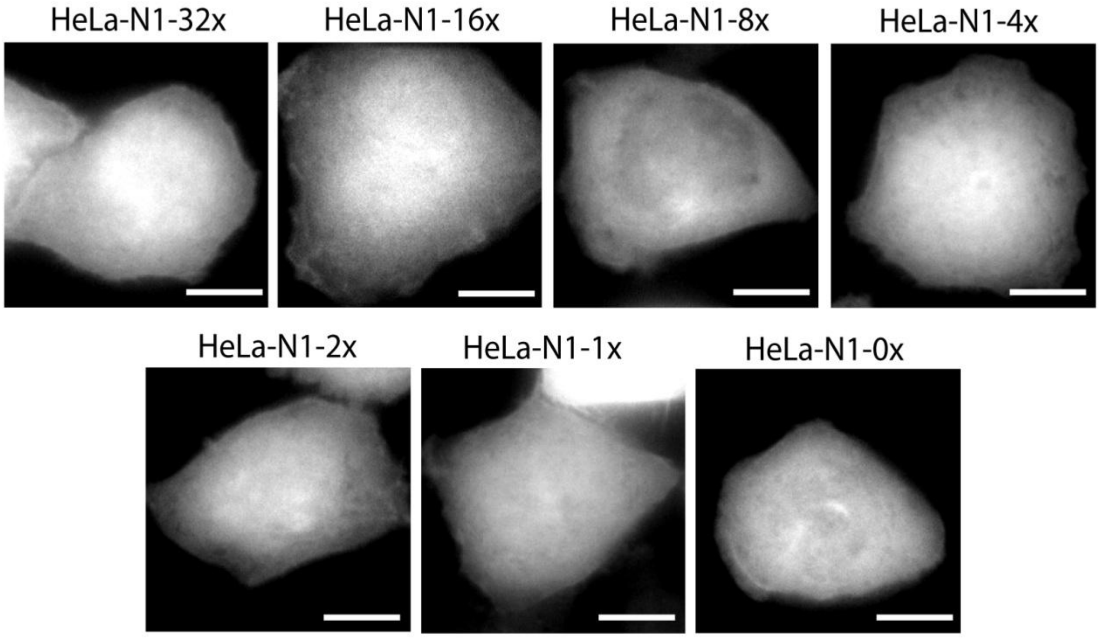

Chen et. al. Supplementary Figure S3

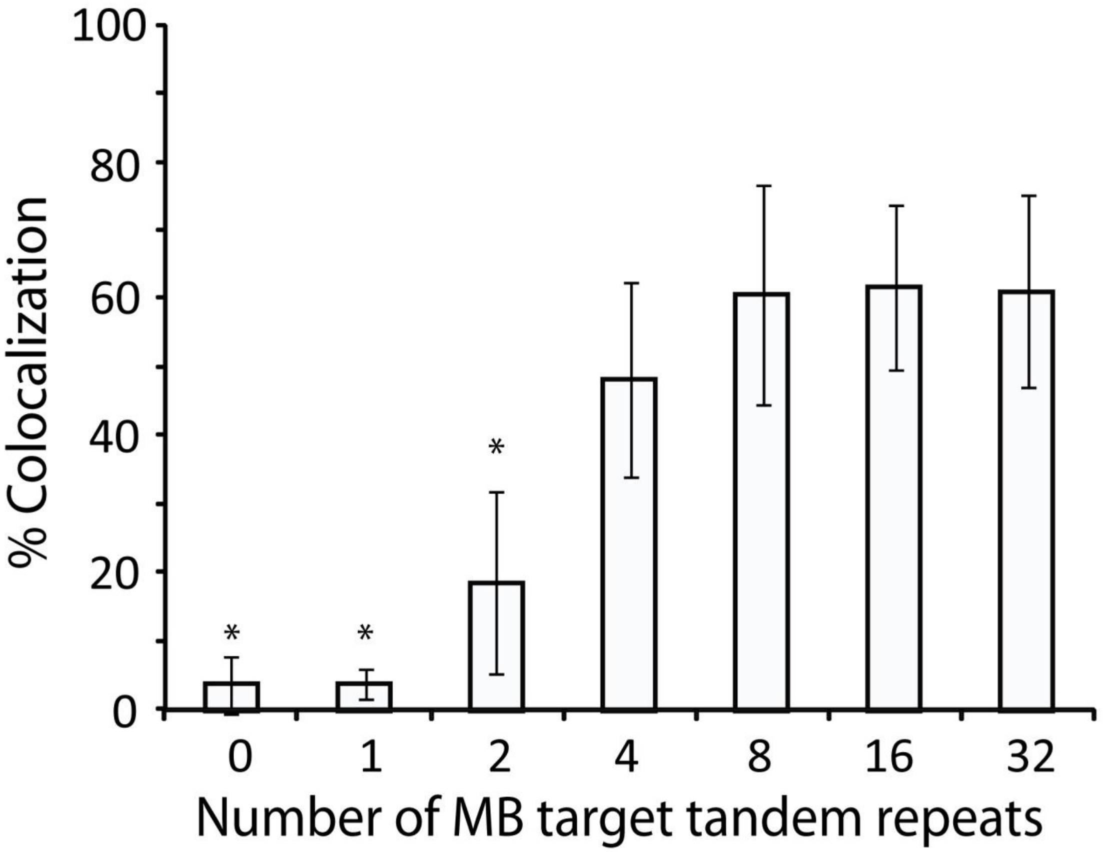

Chen et. al. Supplementary Figure 4

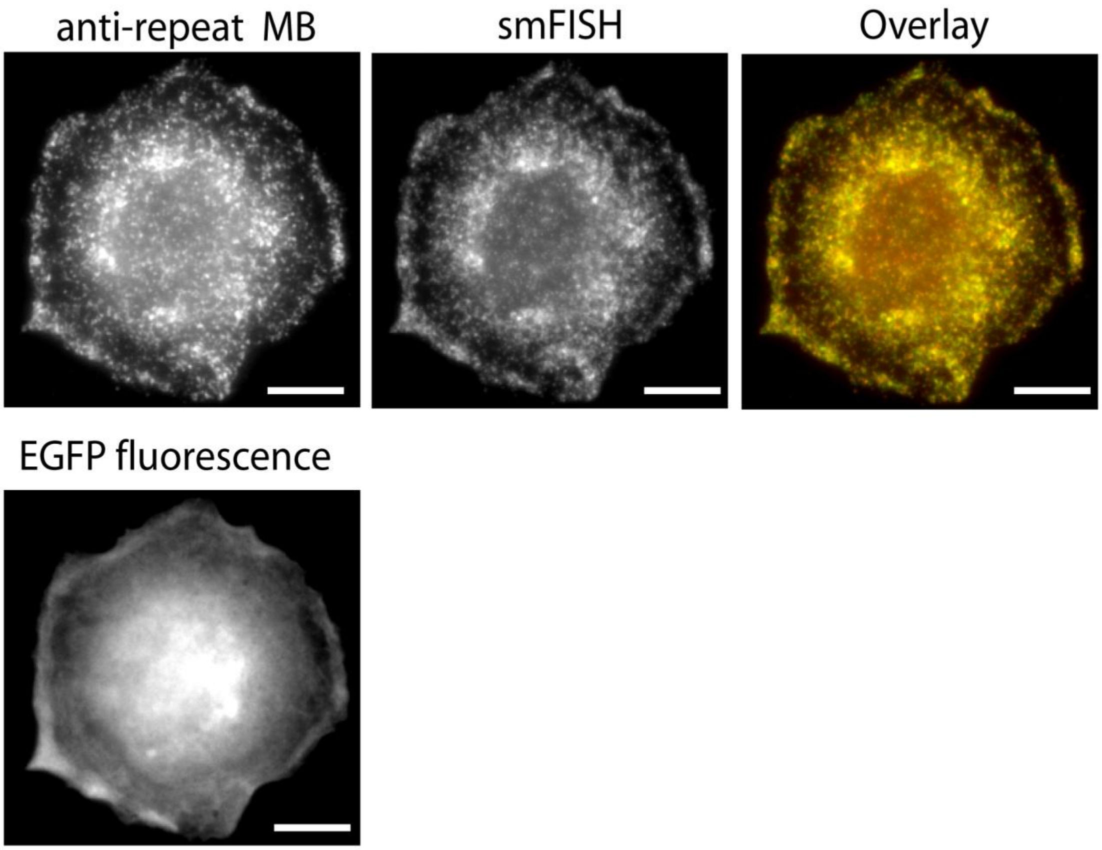

Chen et. al. Supplementary Figure 5

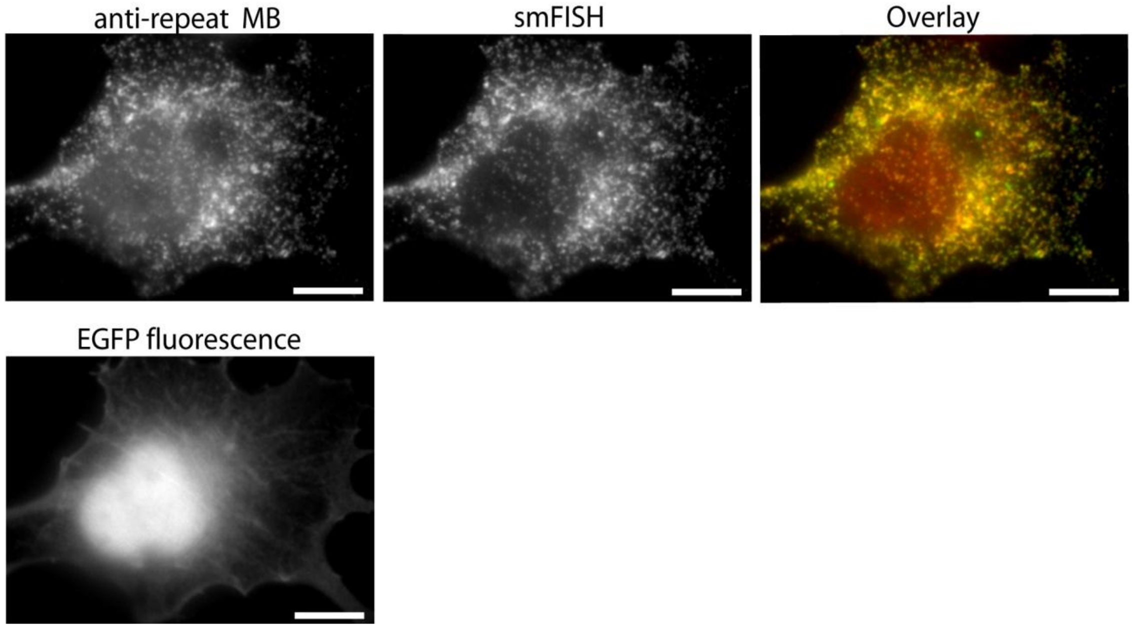

Chen et. al. Supplementary Figure 6

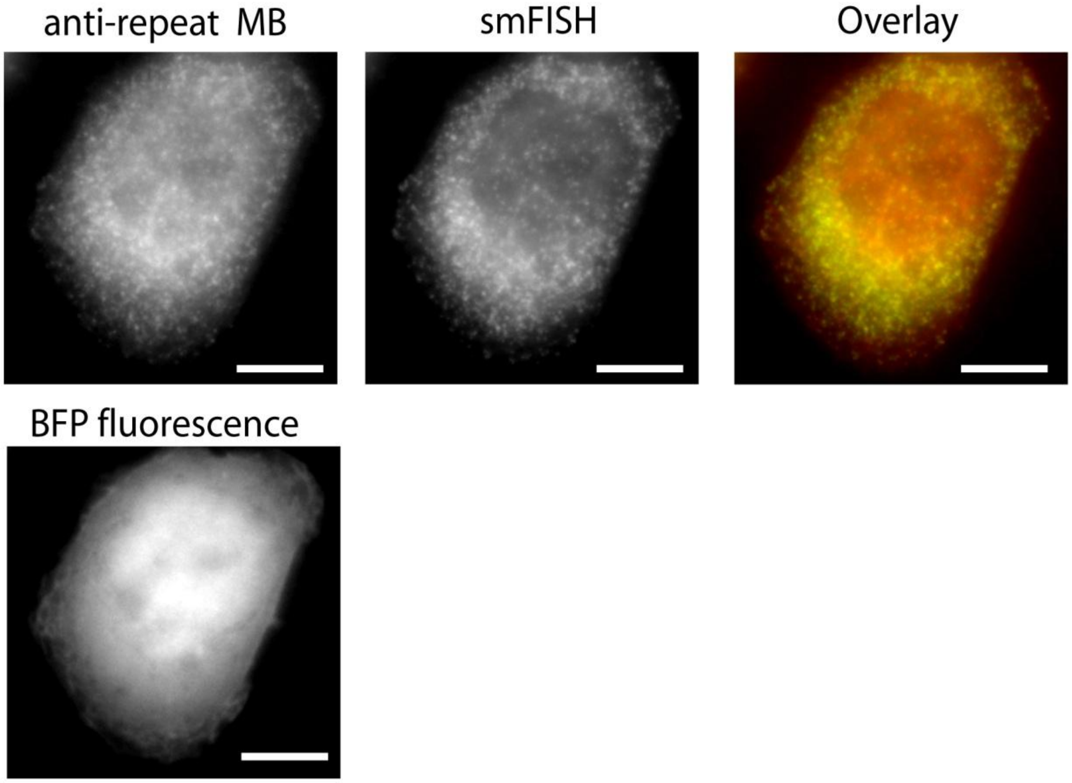

Chen et. al. Supplementary Figure 7

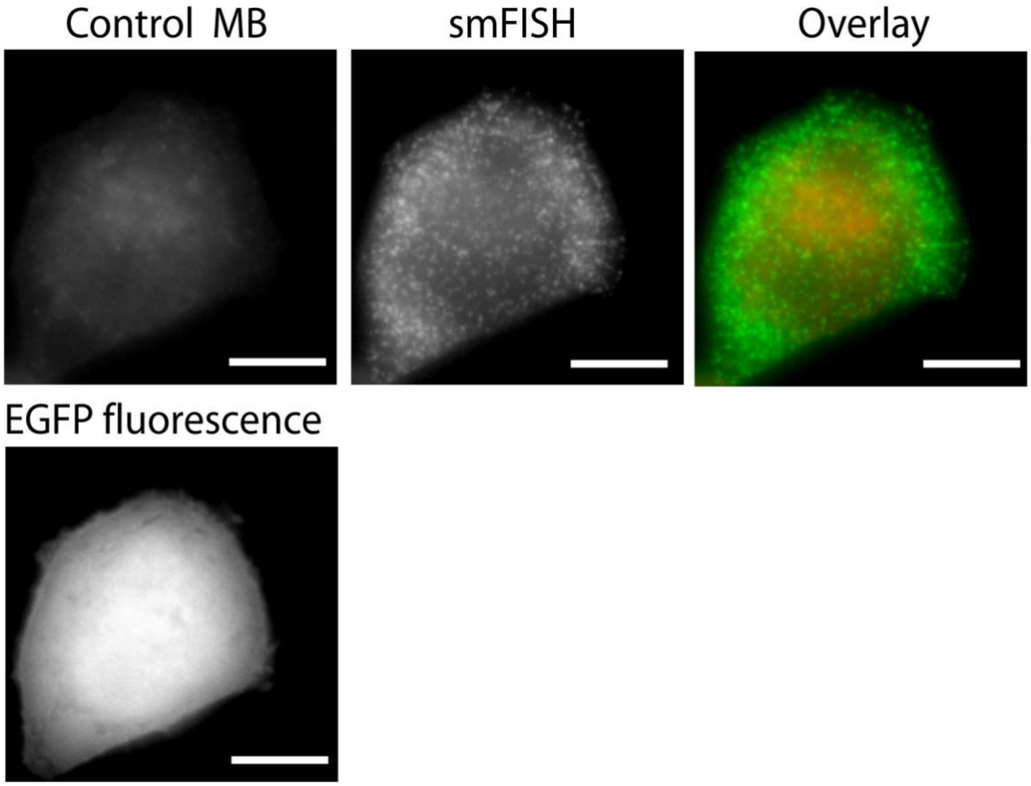

Chen et. al. Supplementary Figure 8

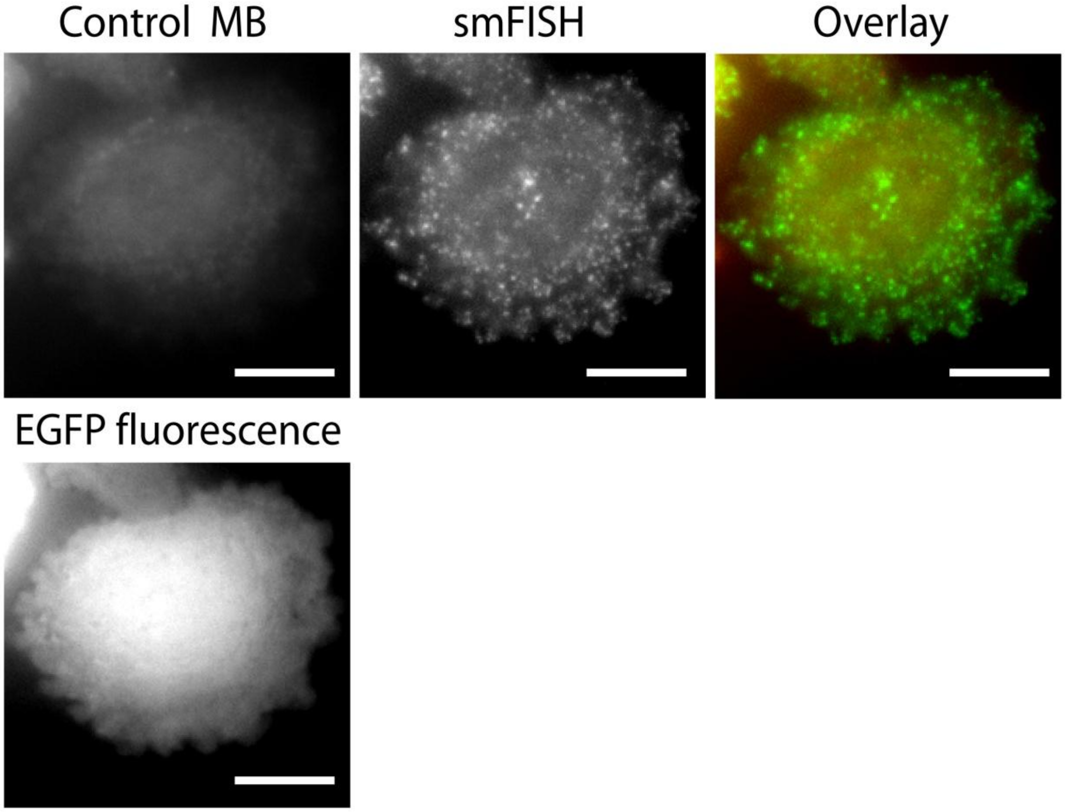

Chen et. al. Supplementary Figure 9

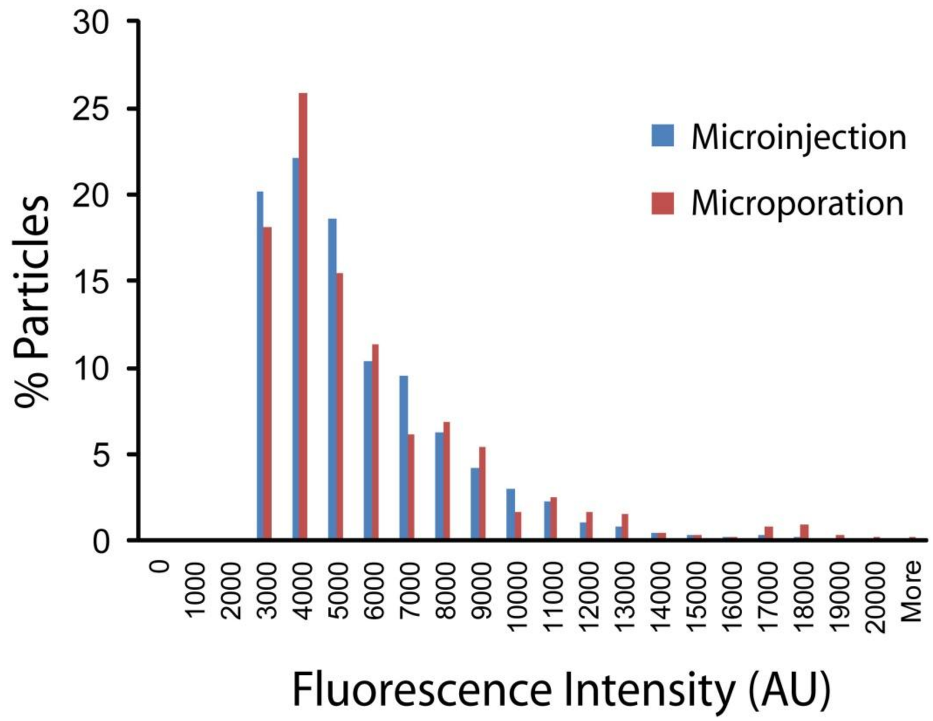

Chen et. al. Supplementary Figure 10

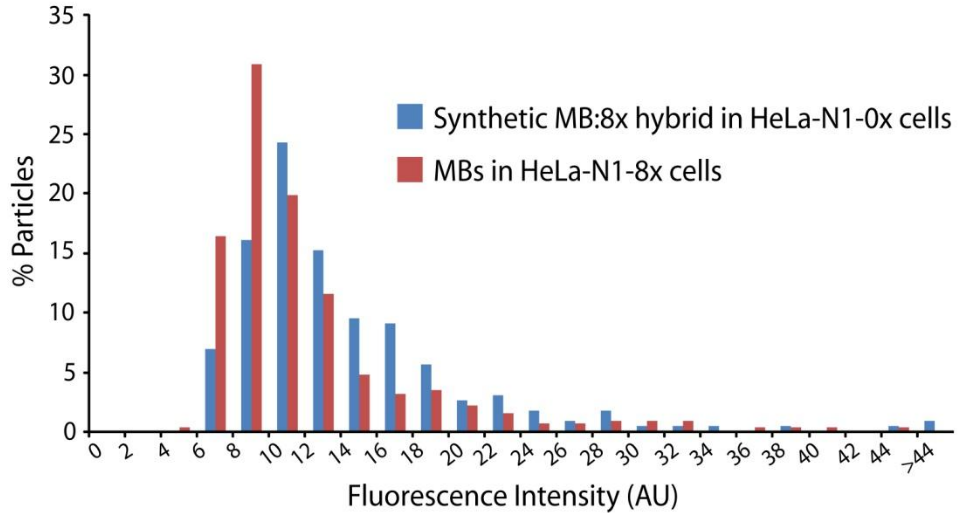

Chen et al. supplementary figure 11.

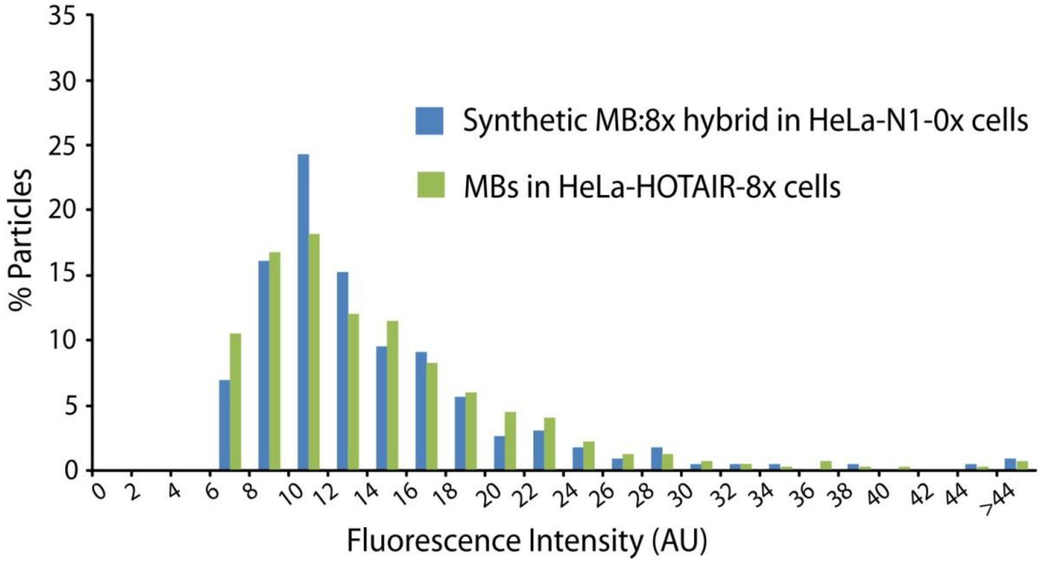

Supplementary Table 1

ttcgcccttagacaccat	gttgtagatgaggcagcc
gcatgttctccttaatca	aagttcacccctctgatc
ctccatgtacagcttcat	atcacagggccgttggat
tgatggttgtccacggtg	agccgagtgttttcttct
cctcggatgtgcacttga	acagcgtctcggtgaagg
ctcgtagggcttgccttc	catgtcgtttctgccttc
ctcgaccaccttgattct	ttgcgatcagatggctcc
aggcgaaggggagagggc	tctatatgtggtcttggc
gctagtagccaggatgtc	gttcttagcgggtttctt
gtcttgctgccgtagagg	tagacgccaggcatcttg
cctgggtgtggttgatga	ttccagtctgtagtccac
gcttgaagaagtcgggga	ttgttggcctccttgatt
gtgaagccctcagggaag	tcgtgctgctcgacgtag
ccgtcttcgtatgtggtg	ctagggaggtcgcagtat
tgggtagcggtcagcacg	aattaagcttgtgcccca

Supplementary Table 2

ctagattttcccttttcctc	agggctggtttcacttttaa
gattattctctctgtactcc	accttcaagagcttccaaag
ttcatgtggcgagctaggac	taacaggcaggtggattcct
taagaagagcaaggaagccc	cactgtgtcttggagaggcg
gcctcatcataaagatggag	ctgccagttagaaaagcggt
agctctctggtcttgttaac	gcatattatagagttgctct
gctgagatagaggtgcttgg	gatgcattcttctagaccta
atcaattaattagcgcctcc	aattaggttacccatgtgtc
cgccatattttacagtccaa	actgcataatcactcctgta
attgtttatgagtccatggg	atttagagtgcaaagtcccg
ttgagagacagtgcactcac	aacttttgtcccaagctggg
tctgacatttgcctatggaa	cctatgtctatttttctact
gatctttccttagcaactaa	gcatcacttatttaagtgtt
ctcctaaaattggtcccatt	ttaaatacccccttctgtgtc
actgaacggactctgtttgg	gtacttctattatttctgtc
ctttggggaagcattttctg	gctgatttttttctgttct
tccaacaaaacacactgcca	ttgggaatggtaatctccat
tttcctataacccaagcttt	atagcaggaggaagttcagg
tgggtctacacaagtagcag	acacaattctctagcaatct
tcgcttccttgtaattctta	aattgcattcttctgggttc
aaattccggagcagctcaag	atacatactgtgtgtgtact
tggacctttgcttctatgtt	atattctgtgagttgtgttt
ctaggaatcagcacgaagca	aaagtgcatacctacccaat
